# Our New York Letter

**Published:** 1891-06

**Authors:** Wm. L. Russell

**Affiliations:** 151 East 50th Street; New York


### Correspondence.

### OUR NEW YORK LETTER.

NEW YORK, MAY 16, 1891.

### TUBERCULIN.

At the last meeting of the Academy there was presented the most satisfactory report of the use of the Koch remedy in the hospitals of this city which has yet appeared. Dr. Kinnicutt, of St. Luke's, read the first paper. He stated that fifty-two cases had been treated at that hospital. Of these thirteen were pulmonary and six laryngeal and pulmonary combined. His experience had taught him that the solution should be freshly prepared at each injection, as the usual carbolized preparation soon deteriorated. The subsidence of the local and systemic reactions, as shown by the physical and general signs, was the best guide in determining the advisability of a repetition of the dose. The action of the remedy imitated admirably nature's method of cure by softening, gradual elimination and sequestration.

He divided his pulmonary cases into three groups. In the first of these local and general improvement occurred, in the second there was no improvement, and in the third decided deterioration. Five cases were included in the first group. Their ages varied from seventeen to forty-two years. In three of them a family predisposition was discovered. In two there had been hæmoptysis. Sputa containing bacilli were present in all the cases, and the physical signs showed infiltration of the apex of one lung. Three cases were under treatment for eleven, thirteen and sixteen weeks respectively, and the maximum doses which they received were sixteen, nineteen and twenty-one milligrammes. There was a gain in weight of fourteen pounds in two cases and of eleven pounds in a third. Two cases were still in the hospital and had gained in weight eight and one-half and four and one-half pounds respectively. The physical signs indicated improvement in the local conditions, and the number of bacilli in the sputa were diminished.

The improvement in these cases had been notable, greater than had ever occurred in the hospital under any other plan of treatment.

Cod liver oil and creosote had been used in connection with the Koch remedy. They had been used alone on the same cases, in one instance for a year, without benefit.

Four cases appeared to be injured by the remedy ; in one an excavation occurred very rapidly after its use was begun.

The impressions which he had gained from five months of observation could be summed up as follows :

1. Tuberculin possessed marked elective tendencies for tubercular tissue.

2. Its value for the purposes of differential diagnosis was relative, not positive.

3. It should be given at first in very small doses, which should be increased gradually.

4. Its use in pulmonary cases should be restricted to the early stage.

5. It was impossible to predict its effect in any case.

6. The constitutional tolerance of different individuals varied.

7. The amount of local reaction was fairly well indicated by the temperature curve.

8. The remedy excited a pneumonic process.

9. Hæmoptysis due to its use was rare.

10. Its use entailed great watchfulness and anxiety on the part of the physician.

11. In the ealy stage of pulmonary tuberculosis it effected a greater degree of improvement than any other method of treatment.

Dr. A. L. Loomis reported briefly the results obtained at Bellevue Hospital. He stated that thirteen pulmonary cases had been treated there. Of these, eight were in the first stage. The general condition of these eight improved, there being an average gain in weight of eight and a half pounds. The number of bacilli in the sputa diminished in all but two cases. The usual result of the treatment consisted of an apparent increase in the activity of the disease, after four to twelve days of treatment; this was followed in about two weeks by a decrease. One case died of disseminated tuberculosis, the *post mortem* revealing tubercles in all the organs. Another became manifestly worse. Of the three cases in the third stage, one died; the others gained thirteen and three-fourths, and nine pounds respectively. There was no change apparent in the cough and expectoration, but in one case the sweats ceased. The conclusions which he had arrived at were:

1. Tuberculin was capable of exciting very active changes.

2 .Its effect depended on the amount given, and on the frequency with which the dose was repeated.

3. If too much was given, rapid extension of the disease resulted.

4. The initial dose should be small, varying from one-tenth to one milligramme.

5. The pneumatic cabinet was a valuable adjunct to the treatment.

6. The number of bacilli in the sputa was no criterion of the effect of the remedy. They might not disappear, even though cure was accomplished. In cases apparently cured by other plans of treatment, bacilli had been found as long as ten years after all other traces of disease had disappeared.

7. In some cases tuberculin aggravated the disease, two cases of general tuberculosis having resulted.

8. Larger and more carefully acquired experience was necessary to secure the safe and proper use of the remedy, but it was destined to take a permanent place in the treatment of tuberculosis.

Dr. N. H. Heineman, of the Mt. Sinai Hospital, believed that the aniline colors would eventually prove valuable adjuvants to the Koch remedy.

Dr. E. L. Trudean, of the Sanitarium for Consumptives in the Adirondacks, stated that eight cases had been treated there by tuberculin. Of these one was cured and one improved.

Dr. Jacobi said that every case must be treated tentatively. Sixteen pulmonary cases had been treated by him, of whom one died, four were not improved, five improved, four improved very much, and two recovered.

Dr. S. Baruch had seen three cases recover under the use of tuberculin, at the Montefiore home. He was unable, however, to attribute any special value to the remedy in these cases.

### ANILINE DYES FOR CANCER.

Dr. Willy Meyer, of the Skin and Cancer Hospital, has been treating a number of inoperable malignant growths with Merck's Pyoktanin, or purified Methylene blue. The treatment was introduced in Germany, where it was discovered that the application of a powder, composed of pyoktanin one part, and talc a hundred parts, was successful in checking the growth of a cancerous ulcer of the breast. Nine cases were treated at the Cancer Hospital, the pyoktanin being applied in the form of powder and ointment of the strength of 1 to 200, and as parenchymatous injections of a solution of 1 to 300, every second day until the fifth injection, when the treatment was discontinued. A maximum dose of a drachm and a half of the solution was innocuous to the general system, the only symptoms observed being nausea, vomiting, weak and slow pulse, headache and general malaise. The local effects were œdema, pain and breaking down of the injected tissue, with perforation of the skin, and discharge. The disease was perhaps not all removed, but much good was accomplished, and this might be improved upon.

### RELATIONS OF NASO-PHARYNGEAL CATARRH TO GASTRIC CATARRH.

At the last meeting of the Academy Section of Pædiatrics, Dr. Fischer read a paper in which he took the ground that gastric catarrh was a frequent result of the presence of naso-pharyngeal catarrh, from the septic matter and alkaline mucus swallowed, and from the continuity of the mucous tract. This was especially true in children under two and one-half years of age, as they did not expectorate.

Dr. Jacobi stated that, although it was true that a close connection existed between the pharynx and the stomach, he had been accustomed to regard catarrhs of both as rather coincident or co-ordinate. They were rather parts of one symptom, the tendency of a mucous membrane being to become inflamed over a wide area. It was possible that pharyngitis might be regarded as a cause of gastritis, but, as a rule, the stomach, accustomed as it was, to the reception of many different substances, some of which were irritating, was perfectly able to deal with the quantity of septic discharge of alkaline mucus which might be swallowed.

Dr. W. P. Northrup has observed that in cases of recovering diphtheria the naso-pharyngeal mucous membrane discharged large quantities of pus, much of which was swallowed, Gastritis was not common in such cases, however, and he was inclined to differ from Dr. Fischer's opinion of the causative relation of naso-pharyngeal catarrh.

DENTITION AS A PATHOLOGICAL FACTOR.

At the same meeting Dr. A. Brothers read a paper on the relation of dentition to various pathological conditions. He stated that formerly all ailments occurring during dentition were ascribed to its influence. Now the pendulum had swung in the opposite direction, and the tendency was to ignore it entirely. He had made observations of five hundred cases, making careful notes of his observations. He had found that the average age at which the first teeth appeared was six and a half months. In a few cases they were congenitally present. Precocious development was not uncommon, and in twenty-one and a half per cent. of the cases the first dentition occurred in the upper jaw. The effect of artificial feeding, wholly or partially, was retarding. In such cases the first teeth sometimes appeared early, but those following were always retarded.

All congenital diseases, such as premature ossification of the skull, heart disease, congenital syphilis, tuberculosis, and deficient mental development and meningitis, caused delay.

Of the diseases acquired during infancy rachitis, it was well known, had a retarding influence. In one-half of his cases with this disease teething did not begin until the child was a year old. Scrofulosis, on the other hand, produced precocity, teeth appearing at an average age of six months. Chronic bronchitis, whooping cough and lung diseases caused delay. Teething was precocious in epileptics, and also in marasmus cases so far as the first teeth to appear were concerned; those coming later were retarded. The diseases which could be traced to dentition were few. He had seen stomatitis thus produced, in some cases pustules appearing in the gum above the emerging tooth. Neuralgia occurred occasionally, but its existence could seldom be determined positively. Fever was seldom due to dentition; in numerous cases in which the child was said to be "fevered," the thermometer showed no change in the temperature. He had never seen meningitis from this cause. He believed that convulsions were a possible occurrence, but only exceptionally. He had never seen paralysis from teething. Blenorrhœic inflammation of the eyelids was regarded by some as an effect of teething; he had not been able to convince himself that this was a fact. Neither had he seen suppurative ear affections from this cause, although Solis-Cohen claimed that one-third of the cases in scrofulous subjects followed teething.

Diarrhœa during dentition was regarded by some as physiological. We know now that diarrhœa is caused by microorganisms in the intestinal canal, and that it seldom accompanies dentition except in the summer months.

Dr. J. Lewis Smith said that he had seen a case in which spasmodic contraction of one set of muscles seemed to be caused by dentition, as it disappeared after five teeth had been cut, and no other cause could be discovered. He doubted if delayed dentition could ever be regarded as normal, and in such a case a suspicion of rachitis should be aroused.

Dr. Jacobi had never seen gastritis result from dentition. He agreed with Dr. Smith that delayed dentition was never normal, but, as in the cases where mixed and artificial feeding were resorted to, indicated a defect in nutrition. He would so regard all cases even when they embraced whole families of apparently healthy children. When teeth were present at birth, it was better to extract them. They usually came out soon and were not settled in the bone, but were merely attached to the connective tissue.

### ATROPINE IN ENURESIS.

In the last number of the Archives of Pædiatrics, Dr. C. G. Kerley, Resident Physician of the New York Infant Asylum, gives some striking results of the use of atropine in twelve cases of enuresis. Nine of the cases were boys and three girls. Their ages were from four to ten years. They wet the bed two or three times during the night, and three of them wet their clothes once or twice during the day also. The custom in the institution is to put the children to bed at seven, and take them up at ten to urinate. No change was made in the case of the children under treatment.

The plan of medication adopted was to put one grain of atropine in an ounce of distilled water. Of this half a drop for every year of the child's age was given at four and at seven P. M. If there were no unpleasant symptoms the dose was increased to one drop. There were slight physiological symptoms of the drug in three cases. After using the remedy for six weeks slight improvement was noticed in four cases ; they went one or two nights without wetting. At the end of the third month these four wet but once or twice a week. Seven were practically well at the end of the fifth month, but the treatment was continued for two months longer, when the dose was reduced one-half. Two months later it was discontinued, and now, nine months after, there had been no return.

The remaining five were slightly improved at the end of the fifth month. During the next three months they continued to improve gradually, and at the end of the eighth month they were not wetting the bed oftener than once or twice a week. During the tenth they wet only occasionally, and the dose was reduced one-half. There was no wetting by the end of a year and the treatment was discontinued. The doctor closes by saying that "eighteen months ago we had twelve bed-wetters of the worst type; to-day they are all well, and the only medicine used was atropia."

WM. L. RUSSELL.
151 *East 50th Street.*

